# Leadless Pacemaker Implantation in a Four-year-old, 16-kg Child

**DOI:** 10.19102/icrm.2020.111002

**Published:** 2020-10-15

**Authors:** Arjun K. Mahendran, Sara Bussey, Philip M. Chang

**Affiliations:** ^1^University of Florida Health Congenital Heart Center, Gainesville, FL, USA

**Keywords:** Heart block, leadless pacemaker, pediatric, permanent pacing

## Abstract

Leadless pacemakers have an accepted role with demonstrable benefits in adults. In contrast, implant experience and follow-up data in pediatric patients are more limited. Clinical indications and patient candidacy for leadless pacing in pediatrics continue to evolve. We present our experience implanting a leadless pacemaker in a four-year-old for the treatment of high-grade atrioventricular block. Implant considerations in small patients, short-term patient and device follow-up data, and follow-up assessment of the vessel used for implantation are discussed.

## Case presentation

A four-year-old male (16.1 kg, 0.67 m^2^) child with a history of heart transplantation in infancy was admitted with a history of unexplained syncope. Historically, despite aggressive immunosuppression, he experienced chronic antibody-mediated rejection and markedly elevated panel reactive antibodies precluded retransplantation candidacy. Long-term treatment has prioritized palliative care and maximization of the quality of life.

**Figure 1:**
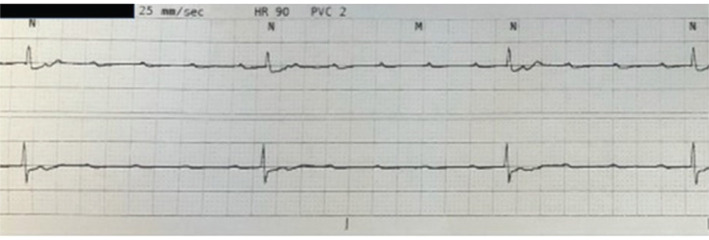
Telemetry-captured second-degree, Mobitz II AV block (transient, complete AV block shown). The patient had normal 1:1 AV conduction before and after the block event.

Inpatient telemetry captured episodes of second-degree, Mobitz type II atrioventricular (AV) block with symptoms of tiredness and dizziness **(Figure 1)**. The episodes generally lasted for several minutes, with spontaneous resolution. Heart block was considered the mechanism for syncope and a sequela of chronic graft rejection. Transthoracic echo showed normal right heart chamber sizes, normal right ventricle (RV) systolic function, and mild left ventricle (LV) dilation with mildly decreased systolic function, all of which remained unchanged from prior imaging. Permanent pacing was indicated and both traditional and nontraditional pacing options were considered. The implications of each approach, including the potential impact on vascular access, the relative expected ease of future endomyocardial biopsies, and postprocedure recovery, were discussed. The parents, transplant team, cardiac surgeons, and electrophysiologist agreed to pursue leadless pacemaker implantation. Given the recent availability of the Micra™ AV pacemaker (Medtronic, Minneapolis, MN, USA), previously approved by the Food and Drug Administration, at our institution, this device was selected for its potential benefit in addressing the patient’s specific conduction disorder.

**Figure 2:**
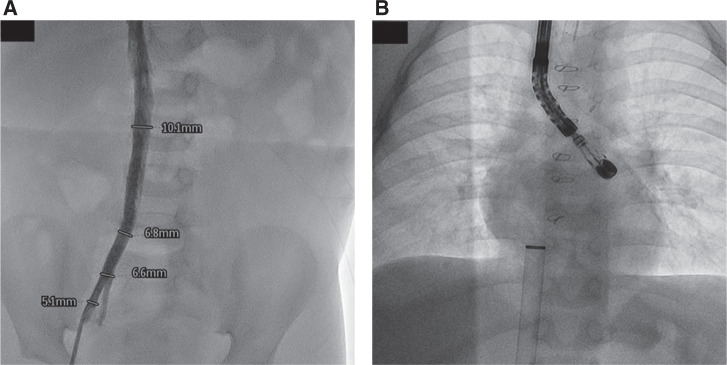
**A:** Contrast injection via femoral venous sheath and measurement of the caliber of the central venous vessels (femoral vein: 5.1 mm in diameter; femoral–saphenous junction: 6.6 mm; external iliac vein: 6.8 mm; lower inferior vena cava: 10.1 mm). **B:** Micra™ IS with radiopaque marked tip positioned in the lower right atrial cavity following serial, progressive dilation of the venous access site.

Conventional right femoral venous implantation was performed under general anesthesia. Ultrasound-guided venous access was obtained, and a 6-French (Fr) sheath was inserted. Contrast injection through the sheath was completed to assess femoral and iliac vein caliber **(Figure 2A)**. The vessels were small but, given their capacity for distension, the decision was made to proceed with slow and serial vessel dilation over an exchange-length stiff wire with a series of dilators. Dilation was systematically performed with 8-, 10-, 12-, 16-, 18-, and 22-Fr dilators. Constant assessments of leg perfusion, skin color, and size were performed during the dilation process. The dilator for the Micra introducer sheath (IS) was used as a final dilator before inserting the complete IS set (27-Fr outer diameter) **(Figure 2B)**. The insertion of the IS proceeded smoothly without resistance.

**Figure 3:**
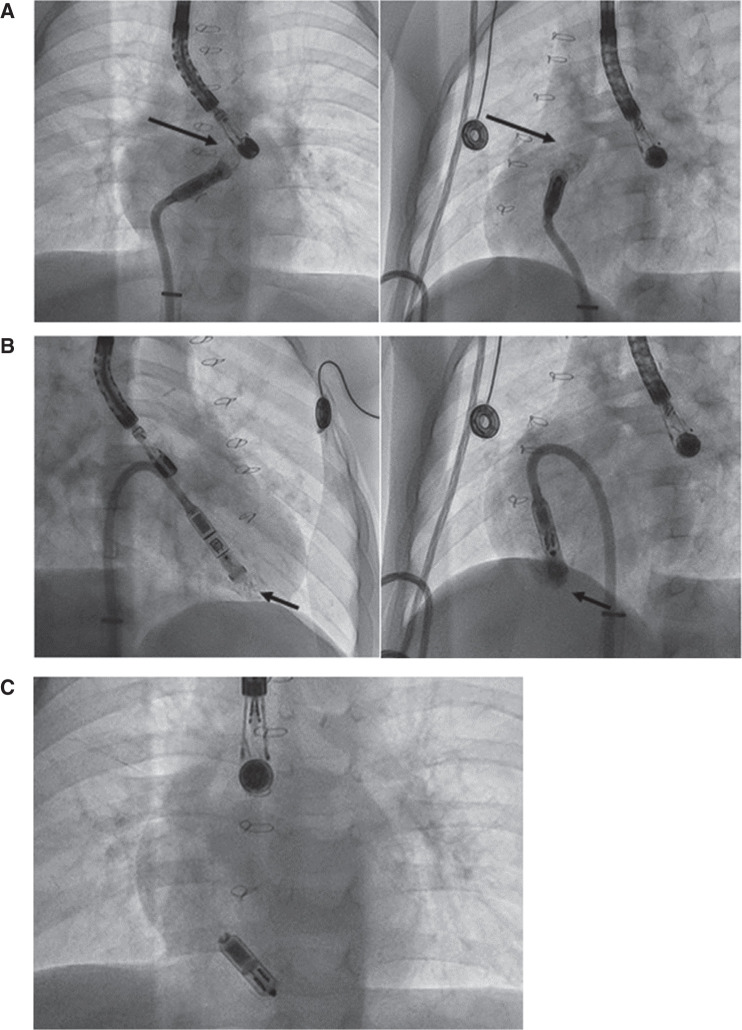
**A:** Attempted midseptal implantation: The DC cup extended toward the outflow tract and pulmonary valve (black arrow). **B:** Apical septal implantation: contrast opacification seen at the septal RV apex (black arrow). **C:** Anteroposterior fluoroscopic projection demonstrating the final implant position of the Micra™ AV device.

**Figure 4:**
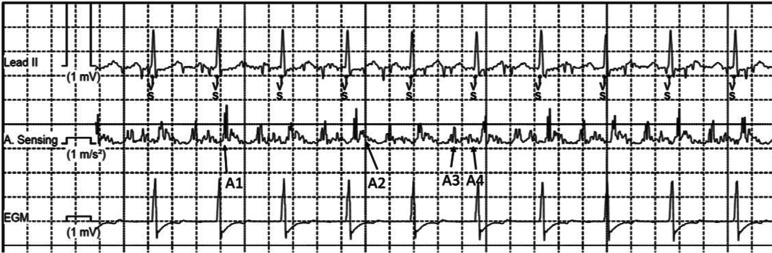
Postimplantation interrogation with assessment of accelerometer signals: sharp A1 and rounded A2 signals correspond to TV (A1) and pulmonary valve (A2) closure during ventricular contraction. Meanwhile, the A3 signal corresponds to passive ventricular filling and the A4 signal corresponds to atrial contraction.

The Micra™ delivery catheter (DC) was prepped and advanced through the IS. To cross the tricuspid valve (TV), active flexion of the DC was performed with counterclockwise torque. Midseptal implantation was initially attempted but adequate septal orientation of the delivery cup could not be achieved. Furthermore, given the dimensions of the chamber, it was felt that midseptal deployment might potentially obstruct the TV apparatus proximally and the RV outflow and pulmonary valve superiorly **(Figure 3A)**. As such, an apical septal location was pursued instead. The TV was crossed again more inferiorly and clockwise torque was applied thereafter to direct the DC tip toward the apical septum **(Figure 3B)**. The device was deployed with at least two splines exhibiting appropriate fixation. Testing demonstrated acceptable functional values (R-waves > 10 mV, pacing threshold: 0.63 V at 0.24 ms, impedance: 570 Ω). Transesophageal imaging confirmed the implant location and showed no pericardial effusion or significant tricuspid interference. The anchoring tether was cut and removed with preservation of the device’s position **(Figure 3C)**. Following DC withdrawal but before IS removal, a 0-Ethibond purse-string suture (Johnson & Johnson, New Brunswick, NJ, USA) was placed around the skin entry site for closure and hemostasis. The site was covered with a pressure dressing. Postimplantation interrogation showed preserved functional values and reasonable accelerometer-derived signals corresponding to atrial contraction **(Figure 4)**.

A right lower-extremity vascular ultrasound performed two days after implantation showed a near-occlusive thrombus in the common femoral vein. The pressure dressing and skin suture were removed on the fourth day after implantation without any evidence of bleeding or hematoma. Given the luminal thrombus, subcutaneous low-molecular-weight heparin (1 mg/kg every 12 hours) was initiated without bleeding recurrence. A follow-up assessment at six weeks after implantation showed normal device function and a reduction in thrombus size with a normal vessel flow pattern on follow-up lower-extremity scanning. By this point, the patient had returned to normal activities without syncope recurrence.

## Discussion

To our knowledge, the present case reports the management of the smallest pediatric patient implanted with a Micra™ leadless pacemaker incorporating VDD mode functionality. The patient’s pacing indication, coupled with avoidance of more invasive implant procedures and complications with leads (transvenous or epicardial) and subcutaneous device pockets, made leadless pacing preferable, especially in light of his long-term palliative care strategy.

Leadless pacing provides effective single-chamber pacing in adults across multiple pacing indications with high rates of implant success, lower complication rates relative to those seen with traditional transvenous devices, and a low infection risk.^1–3^ In contrast, published implant and follow-up data on the use of leadless pacemakers in pediatric patients have been limited, with fewer than 20 total implants reported. The largest single-center cohort to date included only nine patients (median age: 13 years, median weight: 37 kg).^4^ A conventional femoral venous approach was generally adopted, although all patients were older than 11 years and more than 30 kg in weight.^4–6^ Alternatively, Gallotti et al. and Cortez presented different cases performed using right internal jugular venous access in smaller patients.^7,8^ The jugular approach was favored given patient and vessel size considerations as well as the notion of easier device maneuverability across the TV and within the RV from a superior approach. Postimplantation assessment of the vessels used for implantation has not been previously reported.

Our case demonstrates several important considerations to keep in mind when using the conventional femoral venous approach in very small patients. First, slow and serial vessel dilation should be performed. The IS itself, with its hydrophobic outer surface, advanced very smoothly despite the small vessel used in our case and the femoral vessel accommodated the large IS owing to its inherent distensibility. Catheter flexion and device positioning in the RV cavity were easily accomplished from the inferior approach. The application of counterclockwise torque with flexion was extremely effective in crossing the TV. A midseptal implant location for the Micra™ system may not be an ideal choice in small patients, owing to the smaller RV chamber size and potential risks of inflow and outflow interference in these individuals. In addition, adequate septal orientation of the DC tip may not be achievable at this more proximal location on the septum. Postimplantation development of a near-occlusive thrombus in the CFV, while not entirely unexpected, was unfortunate. Although thrombus formation following vascular trauma related to site dilation and large sheath insertion is presumed to be the mechanism, the extended duration of pressure dressing application, which compressed the vessel and slowed venous flows, may also have contributed. It is encouraging that venous patency was preserved with interval reduction of the luminal clot size on short-term anticoagulation therapy.

Although we hoped the VDD pacing capability of the Micra™ AV pacemaker would be useful for our patient’s primary pacing indication, its use in young patients may be limited due to its relatively low programmable upper tracking rate (115 bpm). Mechanical atrial contraction is sensed using the device’s accelerometer and an automated, enhanced sensing algorithm.^9^ This sensed contraction is then used as a surrogate for atrial depolarization from which ventricular pacing can then be sequentially delivered. Given an anticipated low pacing burden and the sporadic nature of Mobitz II AV block in our patient, we were unable to record significant VDD pacing over the short follow-up period. However, manual testing of atrial sensing demonstrated reasonable signals corresponding to atrial contraction and we would anticipate that synchronous pacing can be effectively applied within programmable parameters.

Device retrievability is of particular interest in pediatric patients and has been successfully performed up to four years after implantation.^10^ Given the smaller sizes of RV chambers in pediatric patients, the Micra™ system’s footprint may be proportionately substantial and abandonment of a battery-depleted Micra™ system may limit space for new device implantation. Retrievability was not considered to be a limiting factor in our case given the device’s anticipated long battery longevity in comparison with the patient’s long-term prognosis.

## Conclusion

In summary, conventional femoral Micra™ implantation is feasible even in small patients. The lower limits for age and size for safe and effective Micra™ pacemaker implantation have not been established but appear to be younger and smaller, respectively, than previously published. The assessment of implant vessel size and its capacity for dilation and large sheath accommodation must be performed. Vessel and cardiac chamber sizes can pose limitations to implantation in smaller patients. Synchronous pacing expands the potential use of leadless pacing even in pediatric patients, although upper tracking rates may be a limitation.
